# Knowledge, attitudes and practices (KAP) to assess the impact of school children's awareness of malaria using the MOSKI KIT® tool: study case of some Dakar schools in Senegal

**DOI:** 10.1186/s12936-021-03829-5

**Published:** 2021-07-10

**Authors:** Isaac Akhénaton Manga, Aïssatou Gaye, Aliou Dia, Ekoue Kouevidjin, Maria Rosa Dos Reis, Aboubakry Sadikh Niang, Amy Ndao Fall, Christelle Maitre Anquetil, Jean Louis Abdourahim Ndiaye

**Affiliations:** 1 Department of Parasitology-Mycology, Faculty of Medicine, Pharmacy and Odontology, UCAD, Dakar, Senegal; 2 Medical Inspection of Schools of Dakar, Ministry of Health and Social Action, Dakar, Senegal; 3 Pikine-Guédiawaye Academy Inspectorate, Ministry of National Education, Pikine, Senegal; 4 SANOFI, Dakar, Senegal; 5 SANOFI, Paris, France; 6 Service of Parasitology Mycology, Departement of Medical Biology, UFR Santé, University of Thies, Thies, Senegal

**Keywords:** MOSKI KIT. Malaria, Knowledge, Attitudes, Practices, School children, Senegal

## Abstract

**Background:**

MOSKI KIT® is a fun tool designed to interest children for prevention and management of malaria. This study was carried out with the objective to assess the short- and long-term impacts of this tool on the knowledge, attitudes, and practices of school children, and on the transmission of the knowledge received at the household level as well.

**Method:**

The study took place in elementary schools in the city centre (with relatively low endemicity) and in the Niayes area (at high risk of anopheline and malaria) in the Dakar region of Senegal. The various schools chosen for this study were divided into an intervention group and a control group. The intervention schools were also divided into two subgroups, a full package subgroup and another partial package. During this study three surveys were conducted, the first one before exposure to the MOSKI KIT®, the second one a week later and the third a year later. For the control schools only one survey was conducted and at the same time than the third for the intervention schools. Two household surveys (a week and a year after exposure) were also conducted for the intervention schools against one for the control schools.

**Results:**

Before sensitization, the proportion of school children with a grade above or equal to the average was 50% for the complete package subgroup (CPS) and 53% for the partial package subgroup (PPS). A week later, these proportions were 69% and 71%, respectively for the complete and PPSs. A year later, they were 99.4% for the CPS, 98.1% for the PPS and 99.5% for the control group; The number of children who spoke to their parents about malaria was greater in intervention schools than that of control schools. They were 46.63% and 32.58%, respectively in intervention and control schools.

**Conclusion:**

The MOSKI KIT, has enabled an increase of the knowledge of school children about malaria in the short term and favoured its retention in the long term. However, its impact was not felt on their attitudes and practices.

## Background

An analysis of the data on malaria for the period 2015–2017 highlights the absence of significant progress regarding the morbidity and mortality [1]. The emphasis must, therefore, be on prevention, especially in the context of the absence of an effective and certified vaccine. Raising awareness among children, who are both the most affected and the adults of tomorrow, would be one venue to explore; consequently, training them in prevention is essential. However, children's levels of cognitive and social maturity and their position in society present special issues for the use of community development as a health education strategy [2].

SANOFI initiated the "School Children Against Malaria" programme in 2008 and developed the MOSKI KIT® tool in 2013 to support this initiative. The MOSKI-KIT® is a didactic and fun tool that addresses several themes on malaria, such as: *'How do you get malaria?*' *'How do you prevent mosquito bites*?' *'How to fight against mosquitoes*?' *'How to recognize malaria and treat it*?' The MOSKI-KIT® is composed of the "MOSKI IMAGES®", the "MOSKI CARD®", the "MOSKI GAME®", a "teacher's book", and illustrated wall supports (a weight chart and a poster). It is primarily intended for primary school children, from grade 3 to 6, but can also be used with older children and adults. Its aim is to help teach schoolchildren to recognize and apply the right actions to fight malaria. This kit illustrates the daily life of two children, an older brother, and his younger cousin, in their community. The teaching session on the use of the MOSKI KIT® is divided into 4 stages. The first is an introduction that allows the teacher to explain to the learners the organization and purpose of the session using concrete examples taken from their direct environment. The second stage consists of an animation using MOSKI IMAGES® to encourage exchange and active participation of the pupils. During this stage it is important to ask them to share their personal experience. The third step consists of a validation of the knowledge with the MOSKI GAME®, which uses in a playful way the messages seen during the course. The fourth step is a review of knowledge through the MOSKI CARDS®. The card game is played independently and in small groups. The players must find the messages and talk about them among themselves. These different stages of the use of this tool can be reinforced with the wall display of a weight chart and a poster allowing a daily reminder of the messages in the classroom.

Launched in mid-2013, the MOSKI KIT is currently available in 17 countries (Burkina Faso, Burundi, Cameroon, Ivory Coast, Gabon, Ghana, Guinea, Kenya, Madagascar, Mozambique, Niger, Nigeria, Uganda, DRC, Senegal, Tanzania, Togo). Several evaluations of the MOSKI KIT have taken place to confirm its positive impact on the knowledge, attitudes, and practices of the target audience and trainers. These included those carried out in Burkina Faso in January 2014 with the non-governmental organization (NGO) Edesoin, in Togo in July 2014, with the association Jeunesse en Mouvement pour le Volontariat (JMV), in Benin in August 2014 in collaboration with Aldebarân Foundation, and in Niger between December 2014 and June 2015 with the National Malaria Control Programme (NMCP).

At this stage, 1700 kits have been distributed for specific actions in schools.

Nonetheless its scaling up as a mean of raising awareness among school-aged children is needed. This study was carried out in collaboration with the NMCP, the Medical Division of Schools and a few Academic Inspection of the Ministry/Department Education in the Dakar region in Senegal. Its research question was to determine whether the use of games as teaching tools in schools could have an impact on the acquisition of knowledge and their retention. The objective was to assess the impact of raising awareness among schoolchildren on malaria using the MOSKI-KIT®, but also the relay role that schoolchildren could play within the community.

## Methods

### Study sites

This study was conducted in the Dakar region of Senegal. The region is subdivided into two large zones according to the level of malaria endemicity. A central zone characterized by a low level of malaria endemicity and a Niayes zone which is a flood zone, with a high risk of *Anopheles* development and consequently a year-round transmission of malaria. In each of these areas, three elementary schools were chosen for the conduction of this study. These schools belong to the academic inspectorates (AI) of Grand Dakar in the central zone and Thiaroye in the suburb in the Niayes zone. These schools were selected with the help of the school inspectorates and especially the medical inspectorate of the schools in Dakar, who chose the ones where there were the most children with malaria. In the city centre (Grand Dakar), the study took place in the Imam Abdou Ndiaye 1 and Imam Abdou Ndiaye. In the Niayes area (Thiaroye), these were Martyrs A; Martyrs B; Thiaroye Gare 1 B and Mame Moussé Niang schools.

### Type and period of study

The knowledge, attitudes and practices (KAP) study were carried out in two phases over a period of 13 months between December 2018 and January 2019. We conducted a cohort study in the intervention group in which three KAP surveys were carried out before, 1 week and 1 year after awareness raising with the MOSKI-KIT®. A comparison a comparison of this cohort was made with a group in the third survey.

### Sampling and sample size calculation

The required sample size (n) has been calculated using this formula:

$$n = \frac{{t^{2} p\left( {1 - p} \right)}}{{d^{2} }} = 384$$

Concerning the household survey, all the parents of the pupils who gave a consent for their participation in the study were enrolled.

### Study participants

The school children targeted for this study were those of the grades 4 (G4) and 5 (G5) in intervention schools the first year (2017–2018), and those of G5 and grade 6 (G6) for both types of schools the second year of this study. A questionnaire validated by the director of each school was sent by the teachers to Parents who gave their consent participated to the household surveys.

### Data collection

For the survey conducted before the sensitization and the one, a week later, the KAP data collection, was done on electronic tablets after downloading questionnaires from the Open Data Kit system (ODK). One year after, the survey was done on paper at the teachers request. The same questionnaire was used for each schoolchildren’s survey. For household surveys, questionnaires were administered through phone calls.

### Study description

The different selected schools were divided into two groups: an intervention group (exposed to the MOSKI KIT®) and a control group (not exposed to the MOSKI KIT®). The intervention group was also divided into two subgroups: a complete package subgroup (CPS) (which used all the tools of the MOSKI KIT® i.e., "MOSKI IMAGES®", "MOSKI CARD®", "MOSKI GAME®", the teacher's booklet, and illustrated wall supports) and a partial package subgroup (PPS) (which had used only the MOSKI IMAGES®). Within these schools, different KAP surveys (pre and post-tests) were carried out for school children. The first was a pre-test carried out among schoolchildren in the intervention schools before the MOSKI KIT® was used. The second was 1 week after the tool was used to assess the short-term impact in the intervention schools. The third one year later with the same questionnaire to assess the long-term impact of the MOSKI KIT® in the intervention group compared to the control group.

Household surveys were conducted using a standardized questionnaire to assess the dissemination of school-based knowledge in the community.

### Data analysis

The scale applied to the KAP surveys consists of assigning a score of 1 (one) to each correct answer and 0 (zero) to each wrong answer. The maximum score was 32 (22 in the Knowledge section, 3 in the Attitudes section and 7 in the Practices section). Is considered as correct answer:

- an exact answer checked
- an incorrect answer not checked for multiple response questions
- the total note for a section constitutes the score of the rubric while the total sum of the marks is a overall score of the schoolchildren.

The different data collected had been analysed with the STATA statistical software. To appreciate the impact of awareness raising through the MOSKI KIT®, the proportion of schoolchildren with at least half of the maximum score has been considered. The Chi-square and Fischer tests were used according to their applicability condition for the comparison of proportions.

### Ethical considerations

The study had obtained ethical approval from the National ethics committee for health research in Senegal under the number SEN16/71. Administrative authorization had also been requested from the Ministry of Health and the education authorities in the areas in which the selected schools were located. Parental consent for school children’s participation and interviews was secured before the start of our study.

## Results

### Cohort study

**Fig. 1:**
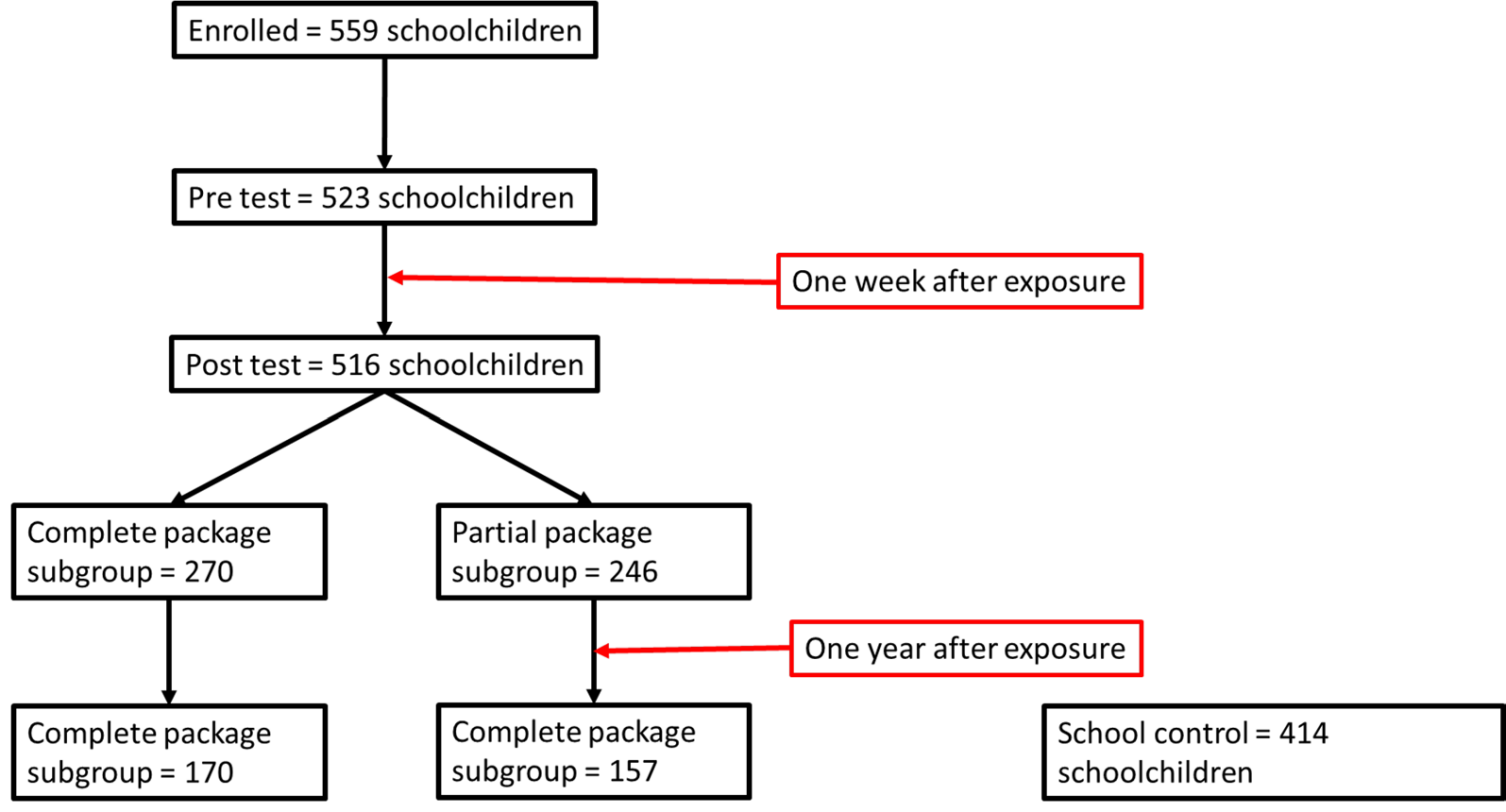
Evolution of the number of school children between the different surveys

In intervention schools, where the cohort study was conducted, 523 school children participated in the pre-test survey with the MOSKI-KIT®. During the second KAP survey (post-test), there were 516 and 327 a year later. In the control schools, we recruited 414 school children. The latter participated in the survey only a year later (Fig. 1).

### Evolution of knowledge

#### Proportion of school children with a grade above or equal to the average in the knowledge section school children

**Fig. 2:**
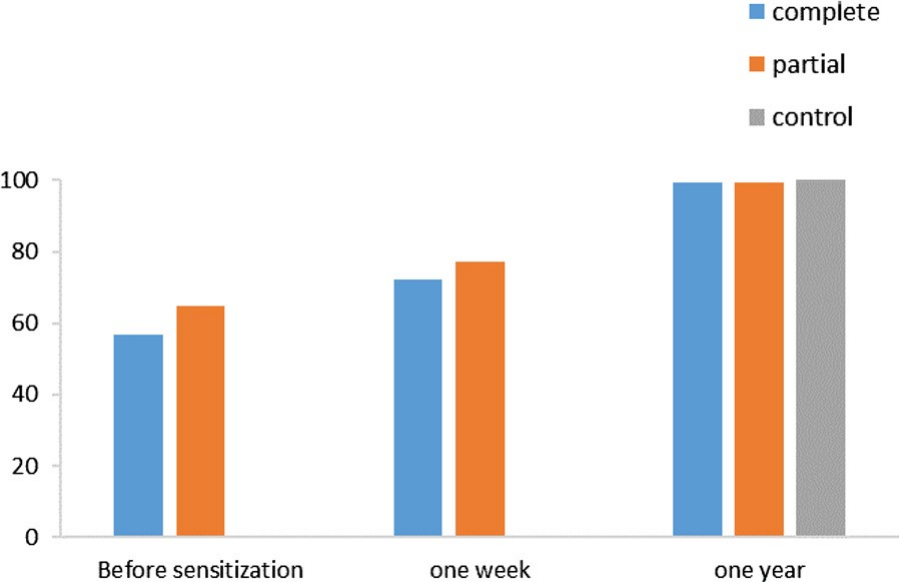
Evolution of the proportion of school children with a grade above or equal to the average for knowledge

Before sensitization, the proportion of school children with the average grade was 57% for the CPS and 65% for the PPS. However, this difference in proportion was not statistically significant (p = 0.07). One week after sensitization, they were 72% and 77%, respectively for the CPS and PPSs. The use of the complete package had no more impact on the increase in knowledge than the use of the partial package of the MOSKI KIT® (p = 0.22). After 1 year, they were 99.4% for the CPS; 99.4% for the PPS and 100% for the control group. No statistically significant difference was noted between the different proportions of the groups 1 year after exposure. (Fig. 2).

#### Difference in proportions of grades above or equal to the average in the two groups of school children in the knowledge section

**Fig. 3:**
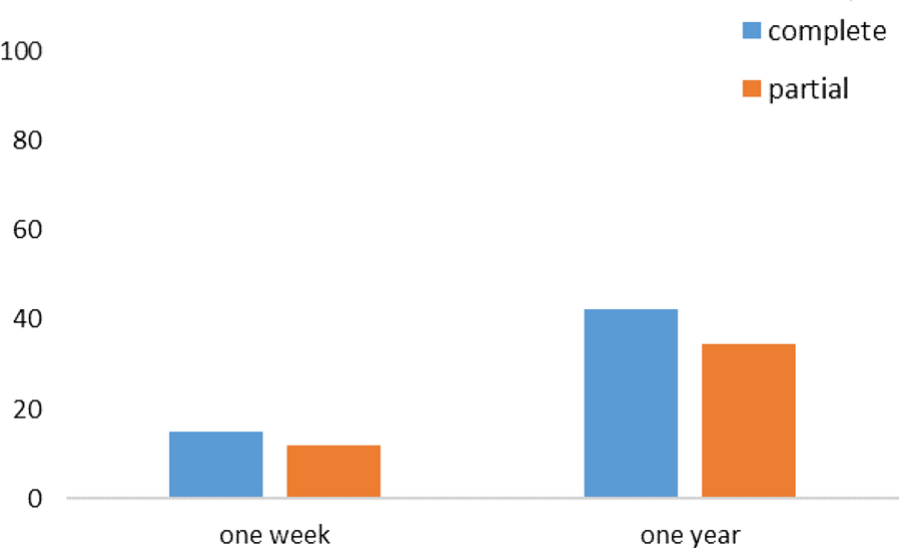
Difference in the proportion of grade above or equal to the average in the two groups for knowledge

The difference in the proportions of school children with a grade above or equal to the average, in the CPS, increased up to 15% and up to 42.4%, respectively 1 week and 1 year after sensitization. In the PPS, it increased respectively up to 12% and up to 34.4%1 week and 1 year after sensitization (Fig. 3).

### Evolution of attitudes

#### Proportion of school children with grades above or equal to the average in the attitudes section

**Fig. 4:**
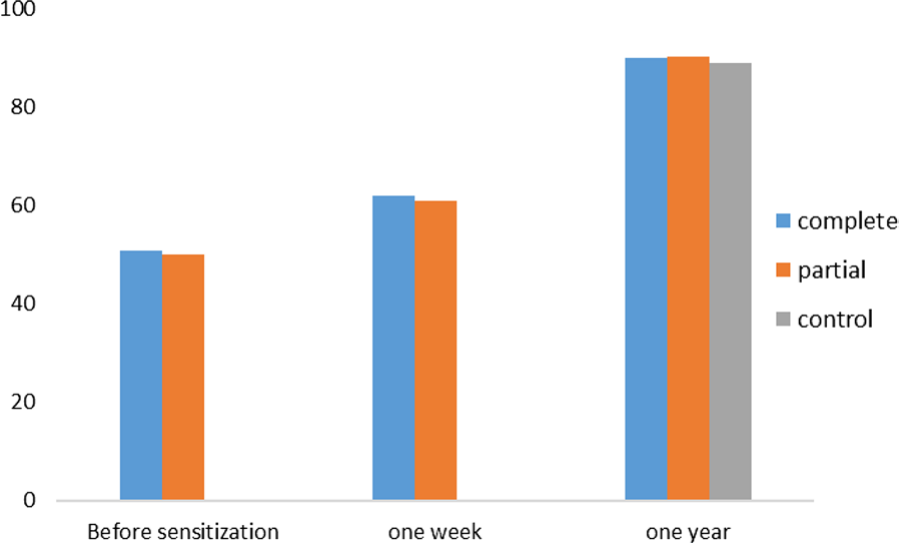
Evolution of the proportion of school children with a grade above or equal to average for the attitudes

Before sensitization, the proportion of school children with grades above or equal to the average was 51% for the CPS and 50% for the PPS (p = 0,90). After 1 week, these proportions were respectively 62% and 55% for the complete and partial package subgroups. An increase in attitudes was noted in the two groups 1 week after exposure. Although this was more important for the CPS, it had no statistically significant difference (p = 0.84). After 1 year, they were 90% for the CPS, 90.4% for the PPS and 89.1% for the control group. No statistically significant difference was found between these groups by comparing them two by two. (p = 1 between CPS and PPS; p = 0.88 between CPS and control group; p = 0,76 between PPS and control group) (Fig. 4).

#### Difference in the proportions of school children with grades above or equal to the average in the attitudes section

**Fig. 5:**
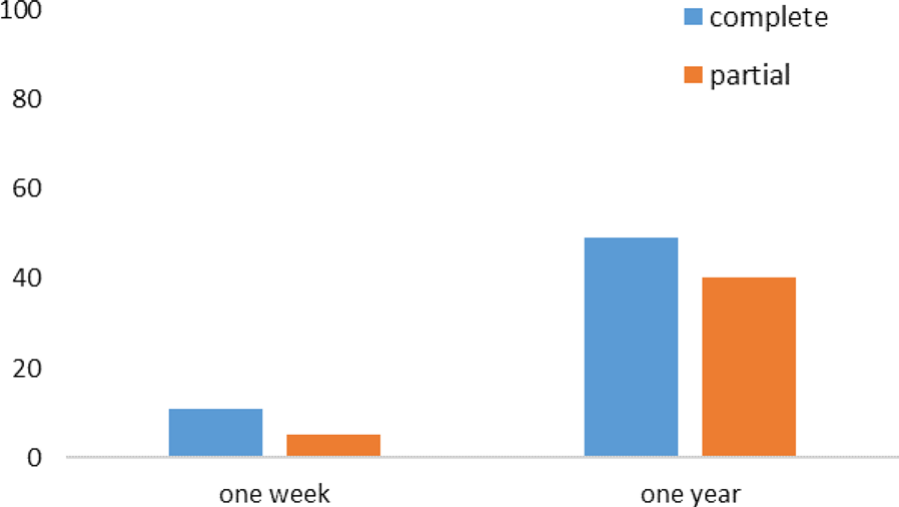
Difference in the proportion of school children with grades above or equal to the average for the attitudes

In the CPS, the difference in the proportion of school children with grades above or equal to the average increased up to 11% and up to 49%, respectively 1 week and 1 year after the sensitization. In the PPS, it increased up to 5% and up to 40.4% respectively 1 week and 1 year later (Fig. 5).

### Evolution of practices

#### Proportion of school children with grades above or equal to the average in the practices section

**Fig. 6:**
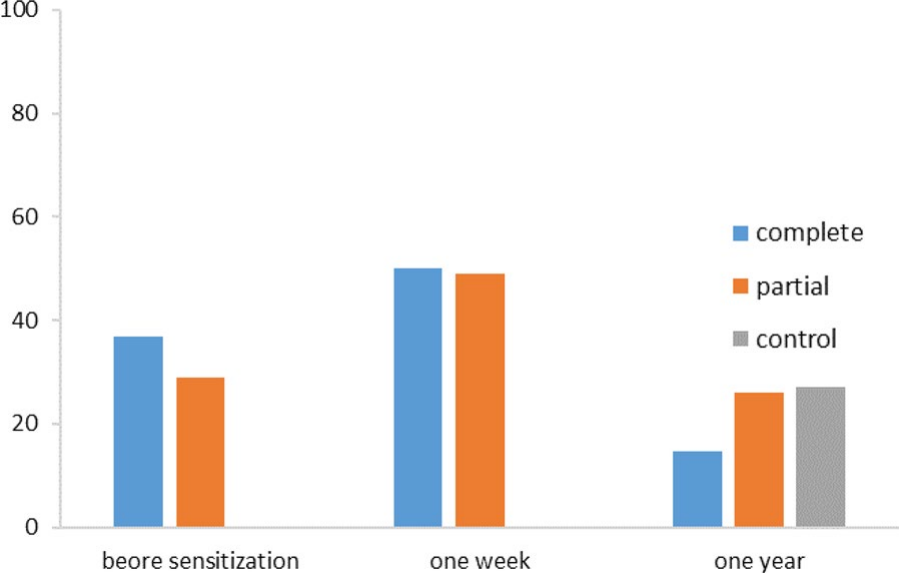
Evolution of the proportion of school children with grades above or equal to the average for the practices

Before sensitization, the proportion of school children with grades above or equal to the average was 37% for the CPS and 29% for the PPS without any statistically significant difference (p = 0.063). After 1 week, these proportions were 50% and 49%, respectively for the complete and partial package subgroups. The increase in the proportion in the practical section was greater for the CPS compared to the PPS, but without any statistically significant difference (p = 0.92). After 1 year, they were 14.7% for the CPS, 26.1% for the PPS and 27.1% for the control group. The proportion of school children with good practices was higher in the PPS compared to the CPS and this difference was statistically significant (p = 0.015). Practices were better in control group compared to the CPS and the difference of proportion was also statistically significant (p = 0.001). On the other hand, any statistically significant difference was noted comparing the proportions of the PPS and the control group (p = 0.244). (Fig. 6).

#### Difference in the proportions of school children with grades above or equal to the average in the practices section

**Fig. 7:**
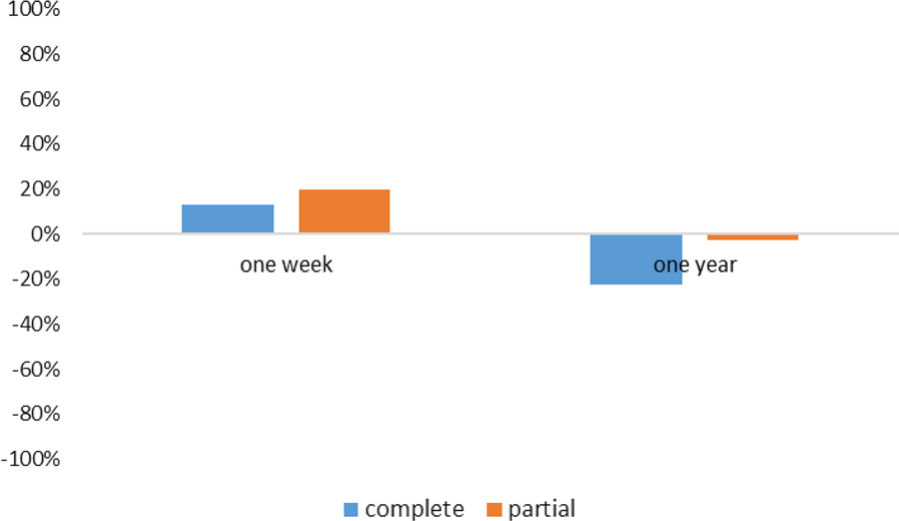
Difference in the proportion of practices with grades above or equal to the average

In the CPS, the difference in proportions increased up to 13% and up to minus 22.3%, respectively 1 week and 1 year after sensitization. In the PPS, it increased up to more than 20% and up to less than 2.9%, respectively 1 week and 1 year later (Fig. 7).

#### Overall evolution of KAP

**Fig. 8:**
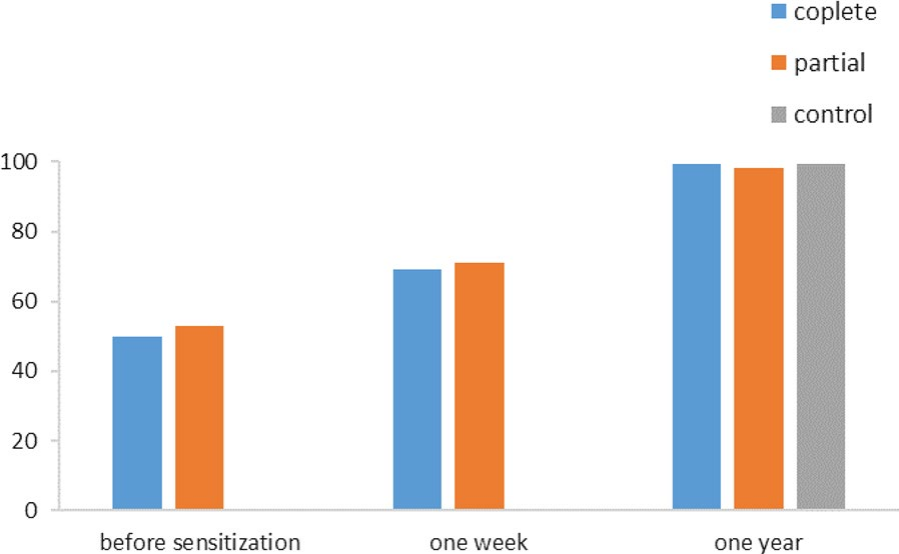
Evolution of the proportion of school children having overall grades above or equal to the average in the KAP

Before sensitization, the proportion of school children with grades above or equal to the average was 50% for the CPS and 53% for the PPS, without any statistically significant difference (p = 0.55). A week later, these proportions were 69% and 71%, respectively for the CPS and PPSs. No statistically significant difference was also noted between these two groups during the 1-week post-exposure survey (p = 0.711). A year later, they were 99.4% for the CPS, 98.1% for the PPS and 99.5% for the control group (Fig. 8). Here too, no statistically significant difference was found with respect to both groups (p = 0.55 between CPS and PPS; p = 1 between CPS and the control group; and p = 0.43 between PPS and control group) (Fig. 8).

#### Difference in the proportions of school children in the KAP

**Fig. 9:**
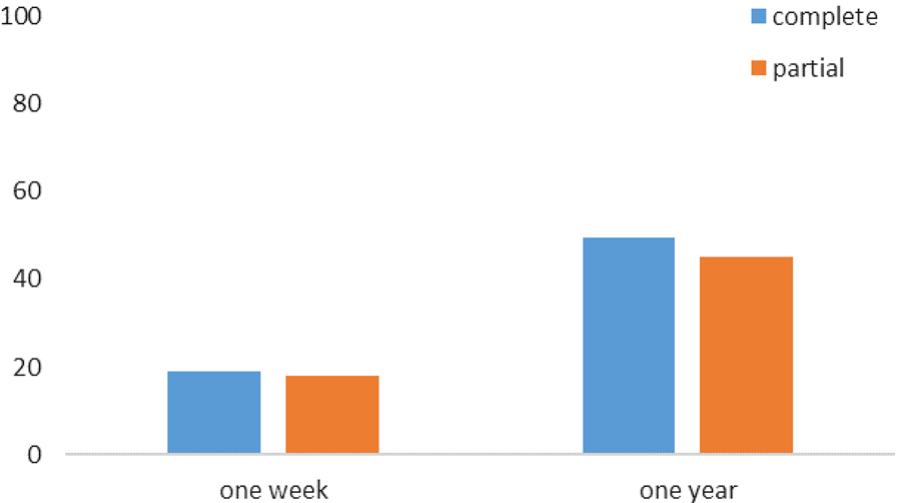
Difference in the proportion of KAPs with grades above or equal to the average

In the CPS, the difference in proportions of school children with grades above or equal to the average increased up to 19% and up to plus 49.4% respectively 1 week and 1 year after MOSKI KIT® sensitization. In the PPS, it increased up to 18% and up to 45.1%, respectively 1 week and 1 year later (Fig. 9).

#### Household survey in the intervention group

**Fig. 10:**
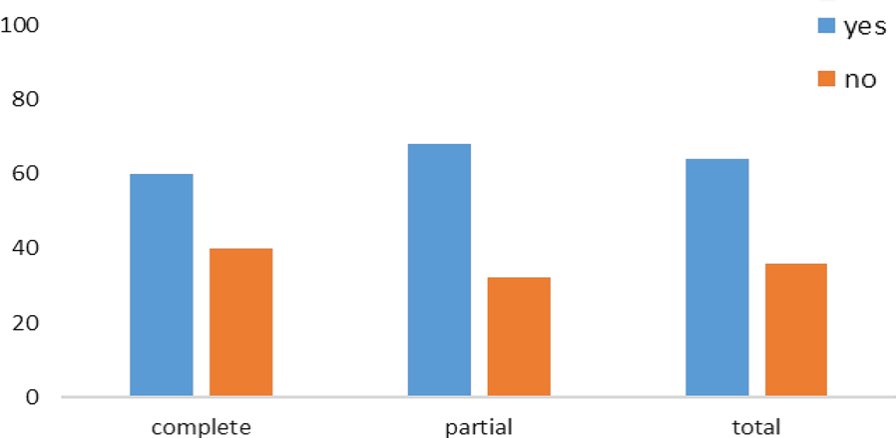
Distribution of parents interviewed according to the answer given and their children's sub-group

Of the 251 parents interviewed, 64% said that their children told them about malaria after the MOSKI KIT® sensitization. Among the latter 68% had their children in the PPS and 60% in the CPS. No statistically significant difference was found when comparing the groups (p = 0.188 between CPS and PPS) (Fig. 10).

#### The themes mentioned by school children

**Fig. 11:**
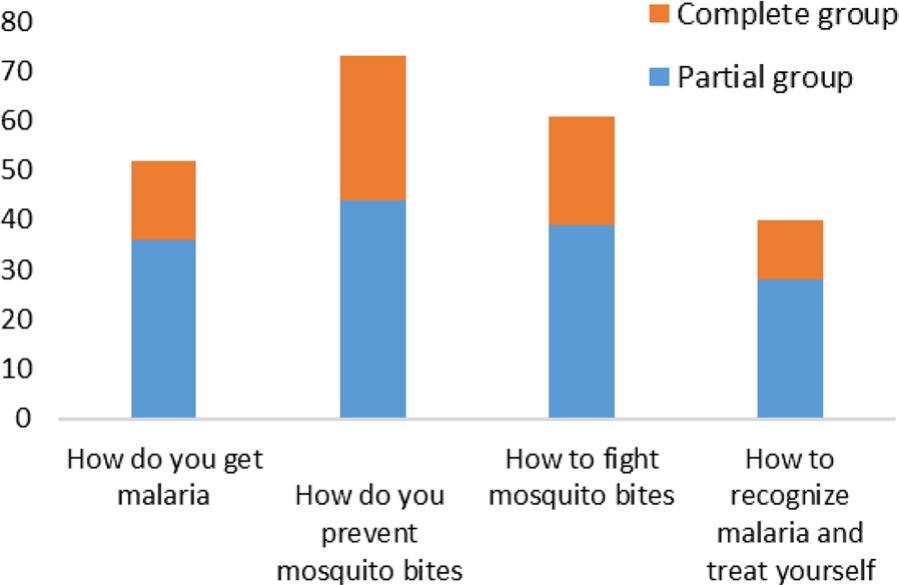
Number of parents according to the themes addressed by school children

Several subjects were tackled by school children at home. Fifty-two parents, including 16 for the CPS and 36 for the PPS, stated that their children spoke about how you get malaria. Seventy-three parents, including 29 for the CPS and 44 for the partial one, said that their children talked about how to fight mosquitoes. Twenty-two parents from the full package subgroup and 39 from the partial subgroup said their children talked about how to fight mosquito bites. finally, 12 parents of the complete package subgroup and 28 of the partial one, affirmed been aware of malaria symptoms treatment (Fig. 11).

During the 1-year household survey after exposure, 268 parents had agreed to participate in this one, 33.21% of whom (95% CI 27.6–39.2%) had their children in control schools and 66.79% (CI 95% 60.8–72.4%) in intervention schools. More children from the intervention schools spoke to their parents about malaria than those in control schools They were 46.63% and 32.58% respectively in intervention and control schools. Exposure to MOSKI-KIT® had an impact on knowledge transfer in families (p = 0.028). About how does one catch malaria? 100% of parents in both schools said that their children had spoken to them about it. With regard to avoiding mosquito bites? 97.56% of parents from the intervention schools had heard about it from their children against 96.55% from the control schools. The exposure to the MOSKI-KIT® had no impact on the return of children in relation to this theme (p = 0.383). Among parents whose children were in intervention schools, 98.73% replied that their children had told them about how to control mosquitoes. This proportion was like that of parents in control schools (96.55%). The exposure to the MOSKI-KIT® tool had no impact on the approach concerning this topic from children to parents (p = 0.466). How do you recognize malaria and treat yourself? The proportion of parents whose children had told them about it was higher in intervention schools (86.21%) compared to that in control schools (67.86%). This difference in proportions was related to exposure (p = 0.045) (Table 1).

**Table 1:** Proportions of school children whose parents answered “yes” to the different questions according to the school

Questions	Intervention schools	Control schools	p
Did your child tell you about malaria?	46,63% (83/178)	32,58% (29/89)	0,028
Did he tell you how you get malaria?	100% (83/83)	100% (29/29)	–
Did he tell you how to avoid mosquito bites?	97,56% (80/82)	96,55% (28/29)	0,383
Did he tell you how to fight mosquitoes?	98,73% (78/79)	96,55% (28/29)	0,466
Did he tell you how to recognize malaria and treat yourself?	86,21% (50/58)	67,86% (19/28)	0,080

## Discussion

This study aimed to assess the impact of games on learning and knowledge retention following the sensitization of school children on malaria with the MOSKI KIT®. Three KAP surveys were carried out a week before and a year after sensitization. An ascending curve in KAP was also witnessed the year following sensitization.

The limits of our study were constituted by the variation in the size of the cohort during the study and the lack of control over other sources of information on malaria. The variation in the number of school children, however, had no statistical impact because the minimum number of participants required was reached.

School children already had a good knowledge of malaria before the intervention. This acquisition could likely be due to the protocol signed in 2002 between the Ministry of Health and Social Action and the Ministry of National Education [3]. These two Ministries considered that it was important for teachers to have a guideline to provide relevant education on the fight against malaria. The guideline aimed at integrating basic knowledge about malaria and current means of prevention into the school curriculum [3]. The “Zero Palu je m'engage” campaign, initiated and led by the Republic of Senegal since 2014, which is based on the fundamental principle that everyone must play an active role in the fight against malaria and get involved to assume this role has also been a contributing factor [4].

Progression in the intervention group was greater in both the short and long term. This increase was much more felt in the CPS. This shows the additional impact of the games. The use of educational and interactive games and cartoons as well are good ways to poke the interest of and to promote long-term information retention [5]. In India, a study had shown that exposing school children to different health education activities, such as live demonstrations, and slide shows when both events were grouped a high average of correct score (47.88%) in comparison to that of control group (26.56%) [6].

Comparing the intervention group to the control one, a year later, no impact of the tool was observed. The lack of difference between intervention and control schools in this study, could be explained by the radio and television programmes on the fight against malaria, broadcast at the request of the NMCP during the period of high transmission. It could also be explained by the lesson on malaria already taught before the surveys.

Raising awareness a week after had a positive impact on practices, regardless of the subgroup. But in the long term, a decrease of these practices, which were not as good in the control group, was observed. It was found that across all groups, practices did not follow the main recommendations of the NMCP [1]. This finding could be explained by the lack of use of prevention tools. In Senegal, a 2017 survey had shown that Dakar had the lowest rates of treated mosquito nets ownership and explained the use of these preventive materials is less envisaged in urban areas because the risk of malaria or the proliferation of mosquitoes is less perceived [7].

A good appreciation of the MOSKI-KIT® and the additional positive impact of games in the knowledge transmission were described by the teachers. The positive role of games in long-term information retention among children had been already demonstrated in an analysis of the literature on the impact of play on learning [5]. The role of relaying information by schoolchildren to their families noted during our study had also been found in studies in Kenya which had shown how action-oriented Child-Child learning methods enabled school children to help peers and parents gain health knowledge that favored changes their practices [8]. Another study in the same country concluded that schools were an effective means of reaching a large section of the population, including future pregnant women and parents of young children [9]. In fact, more than 2500 students from the West and the East of the Democratic Republic of the Congo were made aware of the fight against malaria and then trained to become educators themselves because of strip cartoon entitled "*How to fight against malaria*” [10].

Children and especially those of school age, being the future their participation in the fight against diseases, against malaria, is today an imperative. It becomes then, urgent to implement or disclose strategies intended for them, because of their susceptibility to malaria [11]. These include IPT among school-aged children [12, 13] and educational tools such as the MOSKI KIT®, the MOSKI BOOK® (the world of storytelling for prevention), MOSKI TOON® (a didactic cartoon), which should be added to the prevention and control strategies that already exist.

## Conclusion

The MOSKI KIT had more impact when used with all the tools. However, this impact was not as significant compared to the control group for both knowledge and attitudes. This tool could, therefore, be used to reinforce other existing communication tools for continuous behaviours change for both schoolchildren and the community in the fight against malaria. The use of playful teaching materials in schools could be a good vehicle for raising awareness among schoolchildren and in general populations on diseases and public health problems.

## Data Availability

Data from our study are available and archived at the parasitology-mycology department of the faculty of medicine of the Cheikh Anta Diop University of Dakar.

## Abbreviations

AI: Academic inspectorates
CPS: Complete package subgroup
G4: Grade 4
G5: Grade 5
G6: Grade 6
KAP: Knowledge, attitudes and practices
NMCP: National Malaria Control Programme
ODK: Open Data Kit
NGO: Non-governmental organization
PPS: Partial package subgroup
